# Emphasizing Task-Specific Hypertrophy to Enhance Sequential Strength and Power Performance

**DOI:** 10.3390/jfmk5040076

**Published:** 2020-10-27

**Authors:** S. Kyle Travis, Ai Ishida, Christopher B. Taber, Andrew C. Fry, Michael H. Stone

**Affiliations:** 1 Center of Excellence for Sport Science and Coach Education, Department of Sport, Exercise, Recreation, and Kinesiology, East Tennessee State University, Johnson City, TN 37604, USA; ISHIDA@mail.etsu.edu (A.I.); STONEM@mail.etsu.edu (M.H.S.); 2 Department of Physical Therapy and Human Movement Science, Sacred Heart University, Fairfield, CT 06825, USA; taberc@sacredheart.edu; 3 Jayhawk Athletic Performance Laboratory, Department of Health, Sport, and Exercise Sciences, University of Kansas, Lawrence, KS 66046, USA; acfry@ku.edu

**Keywords:** hypertrophy, strength, training adaptation, sport physiology, sport performance

## Abstract

While strength is indeed a skill, most discussions have primarily considered structural adaptations rather than ultrastructural augmentation to improve performance. Altering the structural component of the muscle is often the aim of hypertrophic training, yet not all hypertrophy is equal; such alterations are dependent upon how the muscle adapts to the training stimuli and overall training stress. When comparing bodybuilders to strength and power athletes such as powerlifters, weightlifters, and throwers, while muscle size may be similar, the ability to produce force and power is often inequivalent. Thus, performance differences go beyond structural changes and may be due to the muscle’s ultrastructural constituents and training induced adaptations. Relative to potentiating strength and power performances, eliciting specific ultrastructural changes should be a variable of interest during hypertrophic training phases. By focusing on task-specific hypertrophy, it may be possible to achieve an optimal amount of hypertrophy while deemphasizing metabolic and aerobic components that are often associated with high-volume training. Therefore, the purpose of this article is to briefly address different types of hypertrophy and provide directions for practitioners who are aiming to achieve optimal rather than maximal hypertrophy, as it relates to altering ultrastructural muscular components, to potentiate strength and power performance.

## 1. Introduction

In most sports, athletes are required to sprint, jump, and throw, (e.g., American football, rugby, baseball) or produce maximal force and power in a very specific manner relative to a competition task (e.g., back squat in powerlifting, overhead press in strongman, snatch in weightlifting, put the shot for throws). Sound strength and conditioning programs incorporate resistance training where power lifts (i.e., back squat, bench press, deadlift) are often used as fundamental exercises to train maximal force production, while weightlifting movements (i.e., snatch, clean-and-jerk, power snatch, power clean) and derivatives (e.g., clean pull, snatch pull, jump shrug, mid-thigh pull) are often used to train power output. During specific training phases, particularly at the early stages of an annual plan, hypertrophy is typically the desired adaptation that can be driven by prescribing the fundamental lifts. Hypertrophic adaptations are ultimately a result of structural (i.e., whole muscle size that can be viewed without a microscope) and ultrastructural (i.e., molecular muscle physiology, visible only with a high-powered microscope) augmentation. The former is often used to determine a positive increase in size via anatomical muscle cross-sectional area (mCSA), depending on how hypertrophy is measured [1]. However, with new evidence emerging, ultrastructural adaptations are often not discussed or considered in relation to hypertrophic training prescriptions specifically for strength (i.e., powerlifters, strongman competitors) and power athletes (i.e., weightlifters, throwers). While the term “hypertrophy” is often used as a general expression of muscle enlargement, there are several forms of hypertrophic outcomes that are possible that will be discussed in detail later in this review. These potential outcomes should be considered when prescribing training volume (i.e., product of sets × reps × load) and intensity (i.e., percentage of 1-repetition-maximum [% of 1RM]) to drive the desired hypertrophic adaptation rather than settling for a general hypertrophic change determined by gross muscle measurements. Additionally, it is vital to point out that hypertrophy and strength are not completely separate phenomena [2], although this is a controversial topic [3]. However, despite the literature supporting or opposing these views, we propose that hypertrophy and strength should be sequentially accentuated so that one training adaptation can potentiate the next (e.g., train to induce hypertrophic increases in preferred muscle fiber size → increase force and power production of the now larger muscle fiber). Therefore, with evidence indicating that preferential hypertrophic adaptations are indeed possible [4,5,6,7,8,9,10,11,12,13,14,15,16,17,18], it may be advantageous for strength and power athletes to increase type II muscle fiber content rather than type I content to improve the associated contractile machinery of type II fibers to potentiate sport performances. Thus, the purpose of this article is to briefly address different types of hypertrophy and provide direction for practitioners to consider when aiming to improve optimal hypertrophy rather than maximal hypertrophy, as it relates to altering ultrastructural muscular components, to potentiate strength and power performance.

## 2. Basic Mechanisms of Hypertrophy

To induce a hypertrophic training response, we must first discuss some of the basic mechanisms and key components associated with hypertrophy. Based on previous literature [19,20], to elicit a hypertrophic training adaptation, the training program should include three different stimuli: muscle disruption [21,22,23], metabolic stress [24,25,26], and mechanical tension [5,27,28], with the latter appearing to be a primary driver of muscle hypertrophy [29]. Most importantly, considering the aforementioned stimuli, protein synthesis (i.e., cells creating proteins) is a key process relative to how much hypertrophy can be gained relative to time. The upregulation of protein synthesis is directly related to mechanical tension by intracellular signaling such as mammalian target of rapamycin (mTOR), mitogen-activated protein kinases (MAPK), and the calcineurin pathway as it relates to calcium flux [30,31]. These mechanically sensitive intracellular signals are also directly involved in mitogenic cellular function and mechano-transduction [30,32,33] mitigating hypertrophic changes. However, muscle disruption is also typically directly related to the stimuli of mechanical tension and must be applied appropriately and repeatedly to engage the muscle tissue remodeling process [34]. For instance, after heavy eccentric focused training [35,36], the extracellular matrix is disrupted due to z-line streaming and microtears within the sarcolemma [37,38] which, in turn, alter the sarcomeres (i.e., the contractile apparatus needed for force production), leading to muscle swelling and soreness when initiating a training program [8]. The muscle remodeling process then releases various inflammatory markers (i.e., interleukins, tumor necrosis factor) to repair the damaged tissue and removes debris (i.e., lactate, H^+^) from the cell [26,39,40]. To aid in the remodeling process, metabolic adaptations may have a synergistic effect through secondary actions such as increased glycogen storage within the tissue [41] or the ability to clear debris at a faster rate [42] to improve the recovery process.

As a result of repeated mechanical tension through training, muscle hypertrophy can occur through a net accretion of muscle contractile proteins along with the regulation of gene expression via messenger ribonucleic acid (mRNA) and microRNA (miRNA) [43,44,45,46]. For example, myogenic miRNAs are in abundance in skeletal muscle mass [47,48]. After receiving an anabolic training stimulus [49,50], a myogenic response can influence how intracellular signals are active to initiate protein synthesis [51]. Each muscle fiber type is capable of resulting in similar hypertrophic changes but may not hypertrophy to the same extent as a result of molecular regulation associated with hypertrophic responses that are dependent on the athlete and training stimuli [52,53]. Gene expression is regulated largely by mechanical signaling associated with the muscle tissue attempting to adapt to the training stimuli through cellular enlargement [54]. This regulation allows various signals to be upregulated and downregulated which can be determined by inducing stretch or force stimuli that can augment gene transcription. Nonetheless, it should be noted that the regulation of muscle growth is likely limited by translation of the messaging proteins (i.e., mRNA). However, miRNAs are small, noncoding fragments of RNA that act as post-transcriptional regulators of gene translation and are an important factor for muscle hypertrophy in strength athletes [43]. Although the mechanisms through which miRNA modulate hypertrophy are unknown, Davidsen et al. [55] suggest that the expression of specific miRNAs via specific training may impact specific muscle hypertrophy and the target tissue [56]. Thus, it appears that the translational process is limited by ribosomal density, which increases during hypertrophic specific training, and may be the rate-determining step in the hypertrophic process. Critical mRNA content peaks after around three to four weeks, playing a vital role in translation and post-translation processes [57]. This event may be different for the upper and lower body musculature and dependent upon sex [58,59]. However, mRNA content may be manipulated if training and appropriately prescribed recovery can upregulate anabolism and protein accretion via the mTOR pathway [60].

A given stimulus must be repeated over time to disrupt fibers through specific ranges of motion followed by adequate recovery periods to result in net anabolism and protein accretion [5,26,61,62]. Once muscle disruption has accumulated, metabolic responses may be altered, resulting in an increased ability to perform work capacity [63]. Metabolic stress may then activate mitogens that produce growth factors (i.e., insulin-like growth factor 1) and mechanoreceptors through specific tension (i.e., maximal isometric force per muscle fiber cross-sectional area [fCSA]), resulting in the activation of mTOR and the gene ribosomal protein S6 kinase beta-1 (p70^S6K^) [35]. However, through the combination of muscle disruption and metabolic changes, mechanical tension must alter the structural and functional component of striated muscle cells that connects the sarcomere to the costamere [64]. The costamere forms a critical component of striated muscle morphology, in that it acts as a locking mechanism by “bolting” sarcomeres to the sarcolemma [65]. Changing the specific tension on the costamere through mechanical stress may allow the integrin associated with Focal Adhesion Kinase (FAK) to activate satellite cell proliferation, promote gene translation, upregulate the ribosomal protein S6 kinase (S6K) pathway, and increase protein translation via mTOR [66]. The mTOR pathway, which is upregulated by S6K and p70^S6K^, is the biological regulator of cell growth which is capable of markedly increasing muscle protein synthesis [67,68]. There is evidence suggesting that mTOR can be selectively activated in terms of upper and lower body musculature [69]. By increasing mTOR signaling responses through a specific training movement (e.g., reach a depth below 90° on back squat), increased mRNA content and muscle protein synthesis in the leg musculature may promote specific and preferential hypertrophy of the leg musculature (e.g., vastus lateralis) which can be transmuted for strength adaptations once the training emphasis is adjusted [69]. However, additional pathways have been identified as particularly important to specific muscle anabolism such as the MAPK and various calcium-dependent pathways. These fellow pathways, particularly MAPK, have been shown to be significantly elevated, albeit acutely, during periods of planned overreaching, intensified training, and high-power training [70,71], similar to what can be seen during hypertrophy training emphases. Many of these anabolic signaling pathways are involved in training-induced muscle hypertrophy, while others function permissively or directly mediate cellular processes that influence mRNA translation stimulating sarcogenesis (i.e., addition of sarcomeres) [26,72]. It is important to note that the number of sarcomeres is not fixed, and is capable of adapting to specific training stressors by increasing or decreasing the physiological amount of sarcomeres [72,73]. For strength and power athletes wanting to improve force and power production after hypertrophy has accrued, sarcogenesis would be considered a positive adaption relative to competition tasks once the muscle has been trained in a competition-specific manner.

Additionally, after a muscle is stimulated by a specific training stimulus, satellite cells (i.e., the stem cells of the muscle that surround the muscle fibers located between the basal lamina and sarcolemma) are primarily involved in muscle maturation, regeneration, and proliferation lasting up to 48 hours post-training [74]. To activate satellite cell proliferation and to elicit the wanted training response via intracellular signaling, special considerations should be applied to the training prescription regarding training volume and training intensity. For strength and power athletes, there are strong relationships between mTOR Complex 1 (mTORC1) activation (i.e., where hypertrophic regulatory actions take place), increased fat-free mass, and strength improvements [75]. For instance, Terzis et al. [75] showed that after 14 weeks of strength training in young males, the phosphorylation activation of p70^S6K^ was strongly correlated with percent increases in fat-free mass (r = 0.81 to 0.89), 1RM back squat (r = 0.84), and type II fCSA (r = 0.82). However, if a hypertrophic training emphasis prescribes high repetitions (e.g., ≥10) per set for prolonged periods (e.g., 6 weeks), the AMP-activated protein kinase (AMPK) intracellular signals may become elevated due to the excessive increase in work that is often dictated by the load used, potentially interfering with the preferential hypertrophic process [76,77]. For strength and power athletes, completing high repetition sets will inevitably lead to training with lower loads. This could, in turn, mute pathways associated with desired hypertrophic adaptations and produce unwanted training effects such as increased type I fCSA [78,79]. In a meta-analysis by Grgic and Schoenfeld [80], it was suggested that high-load training emphasizes type II muscle hypertrophy [81], whereas low-load stimulates greater growth of type I muscle fibers [82,83].

A training adaptation using low loads with higher repetitions, therefore, may also result in mitochondrial biogenesis, leading to a possible shift in myosin heavy chain (MHC) type II (MHC-II) isoform content to be more like MHC type I (MHC-I) isoforms [84]. While this shift is not likely a pure fiber shift initially, MHC hybrid fibers (i.e., MHC-I/IIa, MHC-IIa/IIx, MHC-I/IIa/IIx) may contribute to the observed isoform alterations [85]. However, it has been proposed that hybrid fibers can convert to pure fiber types over time, particularly type II hybrids (i.e., MHC IIa/IIx convert to MHC IIa) [86]. Conversely, preferential hypertrophy could be limited [87,88], due to an unwanted MHC shift towards more oxidative type I fibers [89,90,91] as a result of the inhibition of mTOR signaling [68,92,93] compromising mRNA responses [94]. A MHC shift to more type I-like fiber content would more than likely be disadvantageous for strength and power athletes. These athletes should augment type II mCSA and contractile machinery, while simultaneously decreasing type I content [95] so that muscular adaptations can positively influence improving competition performance outcomes. However, when interpreting fiber type studies, it is important to point out that histochemical misclassifications, particularly when dealing with well-trained muscle, likely take place and go unreported [96]. Therefore, it is important to cautiously interpret fiber type flux findings although, if such fiber type augmentation is possible, positively augmenting the II/I fCSA ratio is warranted.

If training for hypertrophy becomes more of an aerobic-like stimulus from excessive or high amounts of work [97], similar to how bodybuilders train [98], this could lead to a negative cascade of events associated with the AMPK pathway, causing additional issues linked with the upregulation and expression of the myostatin gene (i.e., protein that inhibits muscle differentiation and growth) [99]. Myostatin is primarily produced in skeletal muscle tissues and produced in the blood by binding to a cell-bound receptor that inhibits the action of specific growth factors and mitogens such as growth factors and growth hormone [100]. For animals and humans who lack myostatin expression, muscle hypertrophy is significantly pronounced compared to those with normal myostatin levels [101,102]. This mutation of the myostatin deficiency has been determined to be a result of truncated myostatin mRNA proteins that produce an inactive form of myostatin. However, for most athletes who produce normal levels of myostatin, it may be possible to acutely inhibit myostatin gene expression through a specific training regimen focused on specific hypertrophic adaptations. Once satellite cell proliferation and type II activation take place, the protein regulating skeletal muscle differentiation (i.e., myoblast determination protein-1 [MyoD]) may blunt myostatin activating myoblasts and myogenin, resulting in selective growth of type II fibers [103,104]. Downregulation of myostatin occurs due to an increase in protein myokines such as decorin which bind to myosin heads, prolonging the myostatin response [105,106,107,108,109], and ultimately influencing the molecular regulation and expression associated with the growth processes of type I and type II muscle fibers. Thus, by using appropriately structured hypertrophy prior to strength training foci, the physiological limits placed on muscle growth may be positively augmented to elicit the desired training adaptations and may improve force production and enhance strength potential.

## 3. Structural Skeletal Muscle Hypertrophy

Well-trained individuals often display higher strength capabilities compared to untrained individuals; this is often attributed to the differences in structural skeletal muscle size [110,111,112]. Skeletal muscle hypertrophy is often referred to as a conformational increase to the observed anatomical structure of the muscle tissue, and is often assessed by measuring muscle size via mCSA and muscle thickness (MT) and architectural components (i.e., pennation angle, fascicle length). Monitoring and observing hypertrophic responses are most often undertaken using ultrasound which, within limits [1], is a valid and reliable method for measuring the anatomical mCSA and structure of the muscle [113,114]. The measurement of mCSA is determined by the total cross-section of a particular muscle [114,115,116], whereas MT is determined by the distance between the superficial and deep aponeuroses [117,118,119], both of which are perpendicular to the muscle’s longitudinal axis. These measurements typically provide noninvasive, indirect measurements of anatomical mCSA, with implications for sarcomeres added in parallel (i.e., increased ability to produce force) with observed increases in overall size [117,120,121].

The selected variables of choice to monitor structural adaptations are often mCSA and MT. It is important to note that each component of the muscle architecture is interdependent to some degree. For instance, if anatomical mCSA increases, it is likely that the angle of pennation for that given muscle will likely change with respect to an altered sarcomere arrangement [122]. However, it has been suggested that although an increased pennation angle may be indicative of a greater packing density for sarcomeres in parallel [118,123], there is an established theoretical limit to which the pennation angle may be altered. The pennation angle is a direct measure of the angle at which the fascicles align with the muscle’s line of force production [120,124]. Thus, if the strength training regimen alters the muscle morphology such that vastus lateralis mCSA increases from heavy back squatting, the pennation angle may only change up to the muscle’s physiological limit. Considering that anatomical mCSA has been shown to increase after strength training [125], increasing pennation angle to a point is likely possible with trained individuals. However, the data does not support this notion with untrained individuals [126]. The pennation angle adaptation was previously suggested to range from 0 up to 30°, with 30° being considered the theoretical upper physiological limit for human pennate muscles as related to fat-free mass accumulation [118]. However, it was recently reported that the pennation angle of a multitime world champion, drug-free powerlifter’s vastus lateralis was measured at 33° [122], exceeding the theoretical limit. This may partly explain the lifter’s performance (e.g., back squat 477.5 kgs at the time of the observation [122]). Additionally, this morphological arrangement appears to significantly enable the athlete’s force production capability as it relates to sarcomeres in parallel, which is directly proportional to each unit of force per gram of muscle tissue.

Irrespective of maximal force production, measuring the length of the fascicle is considered a direct measurement of the sarcomere arrangement in series [119,127]. Conversely, increased pennation angle is positively correlated with increased sarcomeres in parallel, which is negatively correlated with the number of sarcomeres in series [120]. However, the angle of fascicles adapts to accommodate hypertrophy within the limited space of the whole muscle which can limit contraction velocity [6,128,129]. While fascicle length is associated with high power, speed sports such as sprinting [119,130], there may be major limits on fascicle adaptations and contraction velocity. Fascicle changes regarding hypertrophy and increases in shortening velocity relate to high power sports such as throwing [131]. For example, Zaras et al. [131] showed that after 25-weeks of sport-specific training, throwers performed better relative to force output and speed as a result of fascicles lengthening and muscle thickness increasing. Therefore, while training may alter a muscle and augment fascicles regarding the sarcomere arrangement, adding sarcomeres in series and parallel relative to the sport could potentially aid in both force and power performances [110].

When dealing with athletes with sporting demands that require rapid force production and high power output, vastus lateralis mCSA and MT are often selected to quantify or predict performance changes [115,117,132]. It is important to note that mCSA and MT often adapt concurrently [133]; thus, these two variables in combination are most often considered and selected for assessing overall muscle size. For instance, in a group of drug-free, national-level powerlifters, Brechue and Abe [118] showed that muscle size was correlated (r = 0.63 to 0.91) with maximal strength regarding powerlifting performance (i.e., back squat, bench press, deadlift). Similar relationships between muscle size with performance data have also been correlated with athletes participating in throwing [131,134,135], sprinting [116,136,137,138], and jumping [115,134,139] events. While the lower extremity musculature is most often used for establishing relationships between observed muscle size and maximal strength capabilities, a few studies have provided correlational evidence for upper body mCSA and MT associated with strength and power performance, particularly with strength and power athletes [122,140,141,142,143]. Aside from correlation data, a recent experimental study by Maden-Wilkinson et al. [110] showed that differences in strength outcomes were primarily explained by differences in muscle size as they relate to stacking sarcomeres in parallel, along with a modest increase in sarcomeres in series with well-trained compared to untrained individuals.

It is well understood that correlational data do not represent nor explicitly show cause and effect relationships between muscle size and strength. However, rather than observing whole muscle size, there is evidence showing that when ultrastructural muscle size is diminished, maximal strength is also reduced with strength and power athletes [144]. Conversely, when ultrastructural improvements are obtained, despite substantial whole muscle growth, maximal strength appears to improve [140,145]. This can likely be attributed to force production at the single fiber level relative to whole mCSA being proportional as observed by Trappe [146]. Thus, the argument for muscle size, often being observed at the macroscopic level, not being related to maximal strength may be misleading, considering that the ultrastructural constituents associated with preferential hypertrophy are often overlooked or not directly considered. Therefore, the following sections highlight the importance of developing and optimizing preferential hypertrophy relative to producing high force and power output by primarily discussing potential ultrastructural adaptions associated with preferential hypertrophic stimuli.

## 4. Myoplasticity and Fiber Type Flux

Myoplasticity has been defined as the capacity of skeletal muscle to alter its structural and enzymatic protein content according to changes in use and the environment (i.e., the training stressor); the effects are predominantly a result of changes in gene expression [147]. It is evident that regardless of training for hypertrophy or strength, if the training volume is increased enough and the intensity is prescribed appropriately, the physiological response may result in hypertrophic adaptations associated with fiber type mutations [148,149,150]. Skeletal muscle in humans is predominately characterized based on MHC isoforms, as previously discussed, categorized as type I, II, and IIX, along with intermediate hybrid muscle fibers such as I/IIA, IIA/IIX, and I/IIA/IIX, with each displaying specific and unique morphological, biochemical, metabolic, and contractile proprieties [151,152,153,154,155]. Regardless of pure or hybrid isoforms, type II fiber content appears to influence whole muscle function, and often correlates strongly with athletic performance associated with force, velocity, and power production [134,140,142,143,151]. The genetic machinery of each fiber type responds to training stimuli by releasing specific mRNA proteins and changing the anatomical mCSA and fCSA, where fibers can take on characteristics of other fiber types (e.g., shifting from MHC-I→MHC-IIA; MHC-IIA/IIX→MHC-IIA) by adapting to the given stressor [87,149,150,156]. However, while fiber shifts may have been observed from type II to I [157], fibers appear to be capable of shifting across the fiber type spectrum predominately taking place within type II fiber content (e.g., IIA→ IIX; IIX→IIA) [155,156,158,159,160]. As mentioned previously, fiber type shift results should be interpreted with caution [96]. For example, in some instances [161], authors have confused fiber type shifts with an increased or decreased amount of MHC content. This does not necessarily result in fiber type shifts, but may rather be indicative of preferential hypertrophy taking place for a type II fiber contributing to a shift in relative MHC expression. Thus, concerning hypertrophying specific tissue and target fibers, it would be advantageous for strength and power athletes to (1) target type II fiber content to increase the type II/I fCSA ratio, and (2) possibly elicit a fiber flux (e.g., shifting from MHC-I/IIA ↔ MHC-IIA ↔ MHC-IIA/IIX ↔ MHC-IIX) throughout the training process, as it relates to specific fitness phases and training timelines, using periodization strategies [162,163].

Training intensity, as previously described as %1RM, alone appears to be a key factor in fiber type alterations [96,160] relative to mechanical stress dictating the extent of cellular transformation, disruption [148], and fiber type II/I content. For a bodybuilder who is concerned with muscle symmetry and shape, training volume would likely be the primary training stimulus rather than high-load, high-intensity training aimed at specifically stimulating type II fiber content for strength or power purposes. Considering that bodybuilders can gain a similar amount of mCSA compared to powerlifters and weightlifters [77,98], bodybuilders are typically not considered strong compared to strength and power athletes; this may be attributed to a smaller II/I fCSA ratio [98,164]. For powerlifters and weightlifters who often train with high intensity (i.e., ≥85% 1RM), low repetitions (i.e., ≤5 repetitions per set) using compound competition movements (i.e., back squat, bench press, deadlift, snatch, clean and jerk), these athletes display 20% greater type II fCSA compared to bodybuilders [85,141]. Bodybuilders tend to train with low intensity (i.e., ≤70%) and high repetitions (≥10 repetitions per set), which may explain why strength and power athletes have muscle fiber diameters twice as large as type I fibers compared to bodybuilders [98,148]. In a review of the literature by Sale [52], indirect evidence was provided for this idea, suggesting that subjects who performed back squats with higher loads showed greater strength gains compared to groups performing movements that only recruited similar musculature with lower loads using leg presses and performing knee extensions. This adaptation was attributed to higher threshold motor unit recruitment and the activation of target muscle fibers.

While training volume has been considered a key mediator of muscle hypertrophy, as noted in bodybuilding style training routines, to acquire critical mRNA content [23], there appears to be a potential intensity threshold associated with whole muscle hypertrophy using a training intensity of ~60% 1RM [26,165]. To target specific fibers and higher threshold motor units, it appears that using higher loads (≥80% 1RM) is warranted for strength and power athletes [136,148]. However, while this threshold is only theoretical, there is likely an intensity continuum that can be used to promote the desired hypertrophic outcome. Considering that muscle force reflects the number of cross-bridges working in parallel, the maximum force developed is related to the fCSA of the specific muscle fiber type targeted. Depending on the number of MHC cross-bridges working in parallel to interact with the actin filaments, the force required to overcome the training stimulus can then be generated at the ultrastructural level. For instance, depending on the training stimuli, protein secretion and MHC adaptations may be different for high-load, low-volume training (e.g., peaking periods) compared to low-load, high-volume training (e.g., preparatory or accumulation periods) [82]. For example, as pointed out by Ogborn and Schoenfeld [82], type II muscle fibers have displayed superior growth after high-intensity strength training [81,129,148,166], yet bodybuilders display greater growth of type I fibers compared to powerlifters as a result of training with high repetitions and lower loads, as pointed out by Fry [148]. It should be noted that the athletes who require great levels of muscular force and power (i.e., powerlifters, weightlifters) are the ones who also possess the greatest content of the fibers capable of producing the greatest force and power [148].

In a recent observation of international level Russian powerlifters, hypertrophic responses were different for training groups using low loads, i.e., 65% 1RM, versus high loads, i.e., at 85% 1RM, indicating that training intensity can dictate hypertrophic outcomes specific to sporting tasks or competition demands [167]. Conversely, Norwegian national powerlifters produced preferential growth of type I muscle fibers (12%) compared to type II muscle fibers (4%) during a blood flow restriction training regimen where the training load remained low (i.e., ~30% 1RM), resulting in less effective hypertrophy [9]. It should be noted that the Norwegian powerlifters also improved front squat 1RM with concomitant hypertrophy; however, it is likely that the strength improvements were more so related to the front squat being a novel stimulus for powerlifters, as noted by the authors, stating that several powerlifters were not familiar with the squat variation and had a hard time keeping an upright torso. Conversely, considering that Bjørnsen et al. [9] provided cellular and whole muscle level muscle hypertrophy measurements, this evidence further supports the notion that muscle hypertrophy was indeed a driver of strength improvements. These observations provide evidence that muscle plasticity gives rise to adaptions that reflect the training stimulus being applied. Understanding the force generation capabilities of each MHC isoform and the impact of these specific adaptations relative to the stimuli, the evidence indicates that there can be a selective hypertrophic response at the cellular level that is greatly influenced by training intensity [9,167]. Thus, for sports with competition tasks that require specific intra- and inter- muscular actions such as high-force, high-power, ballistic movements (i.e., powerlifting, weightlifting, throwing, strongman), increasing specific regional mCSA and the preferred molecular motors such as type II fiber content is warranted.

Aside from directly training for hypertrophy, strength training regimens that are not necessarily focused on hypertrophic outcomes may elicit specific protein turnover for target fibers. Each protein is transcribed (i.e., deoxyribonucleic acid [DNA] to mRNA), translated (i.e., mRNA to protein), and then degraded (i.e., protein breakdown to amino acids) when muscle adaptions occur [168]. During a training emphasis on strength, it is possible to selectively change the relative content of a protein at the ultrastructural level, and its contractile components to take on type II-like properties over time [169,170]. Liu et al. [159] indicated that MHC-IIA fiber content significantly increased with a concomitant decrease in the MHC-IIX content and no significant changes in MHC-I. Additionally, Paddon-Jones and colleagues [171] found a ~15% decrease in type I fibers and an increase in type IIX fibers after six weeks of high speed eccentric resistance training. This adaptation may take place by directly altering the muscle phenotype by augmenting the genetic mechanisms associated with the motor unit to produce more force and power [169,170]. However, the report by Paddon-Jones et al. [171] is problematic, in that the metachromatic staining purportedly identifies four fiber types (i.e., types I, IIA, IIX, and IIC) in adult humans, yet the authors only report three types (excluding type IIC). While type IIX fibers likely contributed to the observed improvements in torque production, these results should be interpreted with caution.

While we understand that the relationship between structural hypertrophy and 1RM strength may not always be perfectly linear [63], increased maximal force of large muscle mass may allow a lifter to become more adept at using the muscle mass recruited for a specific competition task or training movement. As pointed out by Taber et al. [172], theoretical and longitudinal evidence suggest that strength acquisition is enhanced by skeletal muscle hypertrophy. While it is common to see rapid gains in strength early on in young athletes or untrained individuals, Sale [52] indicated that increases in strength are likely due to an optimization of recruitment patterns from the neuromuscular system and associated motor units that may change relative to fiber type characteristics. Using associate movements that are close to the competition motor skill (e.g., powerlifter using a pin squat just below competition legal depth) could be useful to improve competition-specific tasks by promoting an increase in the II/I fCSA ratio by activating the type II musculature and the associated neural components. Theoretically, if a target muscle is hypertrophied, the cellular environment should become larger and increase the muscle contractility potential; however, the contractile changes that do occur may be a limiting factor to enhancing force production. More specifically, mechanical loading can greatly influence the biochemistry of a muscle to enhance the muscle’s ability to produce higher force output [173,174]. Thus, altering the mechanical properties of the muscle as it relates to cross-bridge cycling may further increase the II/I fCSA ratio to improve muscular performance relative to specific tension. Specific tension is a concept that involves maximum force that can be normalized to the physiological mCSA of a specific muscle [175]. It has also been proposed that specific tension plays a role in sarcoplasmic hypertrophy, for example, in bodybuilders where non-force-generating elements are disproportionally increased as a result of hypertrophic adaptations [29]. However, if the sarcolemma enlarges, when accounting for intracellular space relative to the cellular enlargement, it may be possible for myofibrillar function to improve as myofibrillar specific tension changes. Although it may be a null [29] or small contribution [110], increased specific tension could be attributable to changes in neuromuscular activation (e.g., increased agonist activation [176,177]) or an increase in the intrinsic contractile specific tension due to a shift in muscle fiber phenotype [81] or alterations in muscle architecture [178] divergent hypertrophic development.

## 5. Sarcoplasmic and Myofibrillar Hypertrophy

Not all hypertrophy that is developed may be of the same quality or type, or provide the same performance benefits when it is developed. Two different training regimens may be similar in terms of increasing mCSA, but the ultrastructural constituents may be developed differently. One example of this concept is the difference between sarcoplasmic and myofibrillar hypertrophy. Sarcoplasmic hypertrophy has been defined as the chronic increase in the volume of the sarcolemma and related constituents such as the mitochondria, sarcoplasmic reticulum, t-tubules, and sarcoplasmic enzymes (i.e., noncontractile elements) [1,179]. In the early 1980s, sarcoplasmic hypertrophy was mentioned as a potential training adaptation by Stone et al. [63,180] relative to improving anaerobic capacity as a result of high-volume training. Conversely, myofibrillar hypertrophy has been defined as the increase in the size or number of myofibrils accompanied by an increase in sarcomere number or protein abundance related to contractile force generation [179,181]. This has been demonstrated with bodybuilders as well as strength and power athletes [182,183]. It is important to make a distinction between these two modes of hypertrophy, as these variable changes may appear to be of a similar change in muscle hypertrophy via ultrasound (i.e., cellular swelling) [8]; however, the function within the muscle is likely different between these two separate alterations as a result of the training stimuli.

Considering that the force produced by a muscle is related to strongly bound cross-bridging between actin and myosin filaments, the addition of sarcomeres due to myofibrillar hypertrophy should be the desired training adaptation for strength and power athletes, rather than sarcoplasmic hypertrophy [184,185]. Current literature has demonstrated that when examining training-induced hypertrophy between lower repetition ranges (i.e., 6–12) vs. higher repetition ranges (i.e., 15–30), hypertrophic outcomes are similar when examining mCSA via ultrasound or magnetic resonance imaging [23,186]. For example, sarcoplasmic hypertrophic adaptations have been noted in bodybuilders using low load, high repetition training [187] who often train for general increases in mCSA rather than force or power. However, the exact molecular mechanisms underpinning mCSA changes cannot be quantified using gross measurements alone that fail to detect the type of hypertrophy associated with altered cellular size. Considering that direct evidence for sarcoplasmic hypertrophy is expanding [145,182,188,189], albeit remaining controversial [29], a recent investigation demonstrated that after six weeks of high volume resistance training, the major contributor to the observed hypertrophic response was sarcoplasmic alterations [179]. Such adaptations may also increase fCSA while only promoting suboptimal strength improvements. We agree with Jorgenson et al. [29] that the paper by Haun et al. [179], as well as other studies, do not represent the whole population regarding sarcoplasmic hypertrophic adaptations; however, there is still evidence provided indicating that sarcoplasmic hypertrophy may take place, contributing to myofibrillar growth and function [190].

Interestingly, Haun et al. [179] posit that the changes in hypertrophy occur with a decrease in actin and myosin protein concentrations. It is possible that resistance training-induced sarcoplasmic hypertrophy may occur as an initial “precursor” to myofibrillar hypertrophy to enlarge available intracellular space, or as a response to increased metabolism [180]. While it is often thought that sarcoplasmic hypertrophy as a result of bodybuilding style training is associated with nonfunctional adaptions (i.e., increase in noncontractile elements), when accounting for cellular swelling, the force generated by a single fiber is proportional to the increase in fiber size [190]. This theory is also controversial, as it has been suggested that sarcoplasmic hypertrophy does not make a substantive contribution to the mechanical load-induced growth of myofibers [29]. However, with continued training, myofibrillar hypertrophy may occur after attenuation of muscle damage and inflammation in place of sarcoplasmic hypertrophy [62,180], although early-phase fiber adaptations have not previously shown sufficient evidence of edema and fiber swelling, even with untrained subjects [191]. Thus, myofibrillar hypertrophy may not represent a substantial adaptation for several weeks (e.g., >8 weeks) or months after the initial increase in training load [180], although there is evidence to support this notion [145,192,193,194,195], as noted by Jorgenson et al. [29]. Therefore, training should be planned accordingly to ensure that the appropriate stimuli are applied at the appropriate time to potentiate the preferred and desired training adaptations.

When considering the specific type of hypertrophy desired that may be obtained through different modes of training stimuli, an important factor is fatigue associated with training and the possibility of upregulating the AMPK pathway which could, in turn, diminish protein turnover for preferential growth of type II musculature [9,76,196]. Intracellular signals can be muted as a result of chronic training, yet the hypertrophic response seems to reach a peak later in the training regimen, which may be due to residual training effects from inadequate recovery periods [197]. For example, training to failure through various repetition ranges may provide similar quantifiable hypertrophy, yet the fatigue and absolute loading could be different between repetition ranges/outcomes and may diminish gains in II/I fCSA ratio [198,199,200]. Specifically, when training with the lower repetition range, an athlete would, inevitably, handle higher magnitudes of loading compared to high repetitions. Though not directly studied, the different types of hypertrophy through fiber type change, contractile proteins, and sarcoplasmic alterations seem plausible within divergent training modes [180]. For instance, training to failure is often thought to elicit similar or superior hypertrophic outcomes at the structural level; however, the molecular constituents are often not considered. High repetition training or training to failure may not always be desirable when considering sports performance where a balance must be drawn between fitness and fatigue to express preparedness [121]. In fact, a recent series of investigations compared the effects of training to failure versus a nonfailure group [200,201]. The authors demonstrated that compared to the failure group, the nonfailure group expressed greater performance effects and muscular hypertrophy of the type II fibers [200,201]. Thus, although it was not quantified, the recovery cost associated with training to failure could negatively impact the II/I fCSA ratio for strength and power athletes [198,199]. Furthermore, training to failure could lead to undesirable indiscriminate hypertrophy interfering with force and power production capabilities, particularly if the prescribed training is aimed at selectively eliciting regional growth.

## 6. Selective Regional and Indiscriminate Hypertrophy

Due to the ability to alter the architecture, morphology, and fiber type of a given muscle, careful consideration should be given to the development of hypertrophy within a target muscle and across target muscle groups. However, it should be noted that measuring multiple sites of a given muscle to assess regional hypertrophy is critical, given that a single site measurement is likely not reflective of overall architectural differences [110]. This may, in some cases, explain literature opposed to the concept of inducing regional hypertrophy [202]. By examining various athletes’ hypertrophic development in different regions of a muscle, it is evident that across sports, there is a differential need for specific regional hypertrophy to accomplish a sporting task. In sports where relative strength is critical for success, there is a possibility that indiscriminate hypertrophy, where whole muscle growth is realized, may be detrimental for performance. As highlighted in a recent review by Zabaleta-Korta [203], there is ample evidence [12,17,69,204,205,206] suggesting that regional growth of the quadriceps musculature is highly dependent on the exercises prescribed. For example, a sprinter developing distal hypertrophy of the quadricep would likely diminish sprint times due to changing the athlete’s sprinting technique from additional muscle at the knee as a result of indiscriminate growth. Conversely, if the sprinter obtains regional growth at the proximal region of the quadriceps near the hip musculature, sprinting performance could be improved further. The different outcomes in hypertrophy could very well be related to the exercise prescribed and how each repetition is executed (e.g., range of motion, tempo).

Resistance training plans often contain a variegated grouping of exercises that constitute different contraction speeds, types, and ranges of motion, all of which may produce different outcomes when combined or when sequenced together. Some evidence suggests that hypertrophy may be similar between loads and repetition ranges as long as they are taken to failure, yet not all studies agree [199,200]. However, context matters in the argument, and it may not always be possible or prudent to train to failure due to accumulative fatigue of training at or up to the point of failure. Within a given muscle, predictors of force production include both the physiological mCSA and the pennation angle of the muscle [207]. Within the physiological mCSA, it is important to consider shortening velocities as a result of fiber type-specific hypertrophy [208]. If training with specific exercises can develop greater type II fibers compared with type I fibers, there is a possibility of greater force output and shortening velocity resulting in greater muscular power output. When considering pennation angles within muscle, there is an optimal range of pennation angle which may promote force development without limiting the speed of shortening [164,209]. Considering these points, the development of excessive or maximal hypertrophy may result in increases in pennation angle and physiological mCSA that could increase force production at the expense of velocity and limit power output, which is a critical factor for many sports [210]. However, it is still possible for hypertrophy to increase and improve strength, velocity of movement, and power output [211]. This adaptation could be achieved as a result of specific and precise exercise selection.

During the exercise selection process when creating a training regimen, special considerations should be given to the movements prescribed to target regional hypertrophy that can be developed. For example, depending upon the movement selected, it is possible that indiscriminate hypertrophy could alter the moment arm [212]. Depending upon how the moment arm is affected, this could positively or negatively affect how the muscle size adapts [213]. Therefore, based on various joint positions, different contraction types, and contraction velocity, different intra- and inter- muscle hypertrophic responses could be observed. This has been demonstrated in studies where eccentric and concentric contractions were compared and the authors report greater mid-muscle hypertrophy from concentric-only contractions and distal hypertrophy from eccentric contractions [4,69,214]. Within these adaptations, it was demonstrated that the alterations in hypertrophy from concentric actions were a result of pennation angle changes favoring additions of sarcomeres in parallel, whereas eccentric actions also resulted in sarcomere addition in series. This adaptation may be desired for powerlifters wanting to improve force production and weightlifters wanting to improve speed relative to competition tasks. These divergent outcomes may play a role in force generation and shortening velocity, both of which are associated with the development of muscle power output. Biomechanical factors may also play a role in the development of task-specific hypertrophy. For example, in a recent investigation, Kubo and colleagues [215] demonstrated that during 10 weeks of either full or half squats, the group completing the full squat demonstrated greater adaptations in multiple muscle groups compared to half squats. Earlier studies have provided similar evidence that larger ranges of motion result in greater anatomical mCSA compared to smaller ranges of motion in the back squat [216,217]. However, more research should be completed dealing with range of motion and exercise selection, and the question of how, over time, specific exercises and the range of motion may elicit differential hypertrophy depending on the execution of individual exercises. This concept is vital for athletes who need to develop specific hypertrophy relative to task-specific competition movements that require precise ranges of motion for a given lift.

## 7. Optimizing Task-Specific Hypertrophy

Considering the inhomogeneous, indiscriminate changes that can take place concerning whole muscle augmentation, it should be apparent that not all hypertrophy is the same. When attempting to target specific regions of the muscle, the contraction of the muscle displays a large degree of intra- and inter- task specificity. There is evidence indicating that task-specific hypertrophy regarding regions of a muscle is indeed possible [11,12,13,14,15,16,17,18] and should be implemented accordingly. However, using a general hypertrophy or strength-endurance emphasis prior to task-specific hypertrophy (2-4 weeks) may be warranted to initiate the hypertrophy process [81,218], considering that the hypertrophic process can be delayed [219] and the critical mRNA level must be reached. Training prescriptions for bodybuilders typically attempt to increase the overall girth and symmetry of the quadriceps for posing purposes. Conversely, training prescriptions for powerlifters or weightlifters should attempt to increase vastus lateralis and vastus medialis size, as this is related to improved squatting ability and competition performance [220]. Strength and power athletes should be primarily concerned with developing specific hypertrophy relative to (1) the musculature used to perform competition tasks (e.g., pectoralis major and triceps brachii musculature for bench press) and (2) the mechanisms associated with fiber type, the structural components of the muscle, and sporting demands. For example, if a powerlifter or weightlifter accumulates 8 kgs of lean body mass and skeletal muscle mass to fill out their respective weight class and only adds 2.5 kgs to their total, the athlete’s relative competition strength has diminished. However, in this scenario, maximal hypertrophy may have been gained rather than optimal performance related to hypertrophic adaptations.

When optimizing task-specific hypertrophy, special considerations should be given towards increasing the II/I fCSA ratio. For each fiber type, a cross-bridge is the single force generating unit that interacts with thin filaments pulling towards the center of the sarcomere interrelating with specific isoforms which can be altered further through specific training emphasis [221]. Although other proteins and contractile systems are involved with fine-tuning contraction, the prime determinant of force generation is the MHC isoform [222], which is influenced by the training mode [149,223]. Due to biochemical limitations of cross-bridge dissociation, there is a limit on how fast a fiber, and therefore, a whole muscle, is able to contract [224]. There are several muscular structural adaptations that take place due to strength training after task-specific hypertrophy is achieved that aid further in the mechanisms associated with strength changes. Through task-specific hypertrophy, there may be an increased mCSA of the selected tissue with pronounced increases in protein synthesis and remodeling mechanisms that show markedly decreased protein degradation. Specifically, for type II fibers, there appears to be a faster growth rate compared to type I fiber changes, largely due to the less pronounced degradation of type II fibers, partially explaining increases in type II/I fCSA ratio [225,226,227].

Inducing task-specific hypertrophy of synergistic muscles within a group of subregions of a muscle supports the interpretation that neural adaptations, in the form of preferential activation of synergists or muscle subregions in training, are responsible for the regional hypertrophy; this has been supported by EMG and MRI evidence [7,228]. As neural adaptations dominate early training responses, once neural adaptations become asymptotic, the muscular adaptations associated with hypertrophic responses appear to dominate training responses later on [228,229,230,231]. This is important for well-trained strength and power athletes. This may also be influenced by athletes learning a new technique and working on form or finding a range of motion that is best suited to their morphology (e.g., a powerlifter experimenting with different deadlift styles to determine which type best suits limb length). Training at a particular muscle length or joint angle could increase or decrease strength at a point within the range of motion by altering the muscle fiber length, rather than achieving fiber area changes or neural activation [232]. Another possibility is that there is a training-induced increase in the force generated by each myosin head during the interaction with actin; however, there has not been any evidence to support such adaptation. When physiological mCSA increases, it is important to note that although the force generation potential increases, fibers from different motor units are mechanically connected in series and parallel, and activating the neighboring fibers could markedly affect the collective force output attained [233]. Thus, having a larger II/I fCSA ratio within specific musculature is advantageous for strength and power athletes, and may be potentiated further during strength and power training foci [134,142,143,234,235,236,237,238,239].

## 8. Conclusions

It is apparent that skeletal muscle is a dynamic tissue that can adapt structurally and ultra-physiologically to various training stimuli. Rather than achieving maximum hypertrophy, optimal hypertrophy should be acquired relative to (a) increasing sarcomeres in parallel and/or in series relative to predominant competition tasks, (b) increasing the type II/I fCSA ratio, (c) enhancing type II myofibril utility, and (d) accruing select regional areas of hypertrophy to improve the function of type II molecular motors relative to competition tasks. For athletes, the purpose of using specific yet varied training stimuli is to elicit biological changes that could improve performance for a specific sporting task. When considering task-specific hypertrophy for strength and power athletes, heavy loading (80–95% 1RM) while using low repetitions (e.g., 5 repetitions per set) during a hypertrophic emphasis may have greater effects on type II muscular adaptions for strength and power compared to lighter loading [125,240]. Interestingly, to our knowledge, the literature opposed to these ideas has not demonstrated that low-load, high repetition training is more advantageous for improving force production and power output. However, the training volume must be allocated appropriately to elicit the desired structural and ultrastructural adaptations. For instance, by using phase potentiation [218], the previous training phase can promote an adaptation that would correspond to the successive training phase (e.g., improve body composition in phase I prior to specific hypertrophy in phase II prior to maximizing absolute strength in phase III) so that the program is always aimed at obtaining a specific goal (e.g., increasing vastus lateralis and rectus femoris mCSA and MT, improving back squat 1RM, winning a national title) [218]. Considering that task-specific hypertrophy would be aimed at altering the sarcomere arrangement and other ultrastructural constituents, if a muscle is not habitually trained through the fullest range of motion or a specific range of motion, then negative muscle alterations may arise, reducing force and power capabilities. This concept is particularly important, considering that muscle fibers are capable of monitoring sarcomere changes through an array of stimuli, resulting in the addition or removal of a given muscle unit [31], and may also be attributed to the fiber type flux. Thus, a task-specific hypertrophic emphasis appropriately applied early on may potentiate strength and power adaptations, training, and performance if the target musculature is enhanced at both the structural and, more importantly, the ultrastructural level.

Strength and power athletes are considered a unique cohort of athletes; yet, all athletes, to some capacity, regardless of sporting discipline, must train for metabolic adaptations, work capacity, hypertrophy, strength, and/or power at some point within their athletic career to enhance performance. For strength and power athletes, it is feasible to suggest that augmenting specific hypertrophic adaptations prior to sequential strength and power training foci would appear to facilitate and potentiate strength and power capabilities further. For instance, a powerlifter may wish to increase specific quadriceps, triceps, chest, and back musculature relative to how the athlete squats, benches, and deadlifts for force purposes. Likewise, a weightlifter may desire to increase specific quadriceps, shoulder, and back musculature for velocity, as well as force, relative to how the athlete executes a snatch and clean-and-jerk. Throwers may focus on specifically increasing sarcomeres in series in the upper extremity for velocity and power purposes, along with sarcomeres in parallel in the lower extremity for force production. Conversely, a strongman competitor may seek to obtain hypertrophy juxtaposed to the thrower, as competition tasks often require high force output for upper extremity musculature (e.g., log press) and somewhat velocity-based tasks while under heavy load for the lower extremities (e.g., yoke walk, farmer carry). Nevertheless, we suggest that all of the aforementioned athletes, and others involved with strength and power events, should attempt to improve type II musculature and the molecular motors therein.

Considering these points, we disagree with research groups who have considered the relationships between hypertrophy and strength extraneous [3], considering that size and strength are critical for strength and power athletes. We hope to have provided evidence of how training for task-specific hypertrophy can result in an array of hypertrophic outcomes, and is unique to sporting demands and competition tasks. We urge researchers to test these concepts by using ultrasound, biopsy measurements, and 1RM testing when assessing skeletal muscle adaptations relative to strength training. Based on the evidence provided, we conclude that structural and, most importantly, ultrastructural training adaptations because of task-specific hypertrophic stimuli could contribute to competitive athletic strength and power performance, particularly for strength and power athletes involved in individualized sporting events.
